# A Flexible Miniature Antenna for Body-Worn Devices: Design and Transmission Performance

**DOI:** 10.3390/mi14030514

**Published:** 2023-02-23

**Authors:** Abdullah Al-Sehemi, Ahmed Al-Ghamdi, Nikolay Dishovsky, Nikolay Atanasov, Gabriela Atanasova

**Affiliations:** 1 Research Center for Advanced Materials Science (RCAMS), King Khalid University, Abha 61413, Saudi Arabia; 2 Department of Physics, Faculty of Science, King Abdulaziz University, Jeddah 21589, Saudi Arabia; 3 Department of Polymer Engineering, University of Chemical Technology and Metallurgy, 1756 Sofia, Bulgaria; 4 Department of Communication and Computer Engineering, Faculty of Engineering, South-West University ‘Neofit Rilski’, 2700 Blagoevgrad, Bulgaria

**Keywords:** flexible antenna, miniature antenna, off-body wireless communications, in-body wireless communications, SAR, polymer substrate

## Abstract

The last few years have seen a rapid increase in body-worn devices because these devices cover a broad spectrum of potential uses. Moreover, body-worn devices still require improvements in their flexibility, size, and weight that necessitate the development of flexible and miniature antennas. In this paper, we present a new flexible miniature antenna for body-worn devices. To ensure flexibility and comfort when the antenna is in contact with the human body, a substrate from natural rubber filled with TiO_2_ is developed. The miniaturization is achieved using the quadratic Koch curve. The antenna design, optimization, and characterization are performed on a human body model. The performance of the antenna is analyzed in two scenarios: (1) in- to on-body, and (2) on- to off-body wireless communications. The results show that the antenna realized the maximum telemetry range of more than 80 mm for in-body communications and more than 2 m for off-body communications. Moreover, the highest 10 g specific absorption rate value was 0.62 W/kg. These results, in addition to the antenna’s compact dimensions (12 mm × 26 mm × 2.5 mm) and the low manufacturing price, make the proposed antenna an ideal candidate for health telemetry applications.

## 1. Introduction

The interest in body-worn devices has been growing steadily in the last few years because they have found applications in many areas [1,2]. Statistics [3] show that, during and after the COVID-19 pandemic, applications for remote monitoring of human health using body area networks (BANs) demonstrated an acceleration in growth. The application of wireless networks for animal health monitoring has also increased in the last few years. With increased use, there is growing research interest in body-worn gateway devices, as they provide wireless communication across an implanted device, a body-worn device, and an external device using channels in, on, or outside the body [4,5,6,7,8].

One of the primary elements of any body-worn gateway device is the antenna [9]. Many challenges are associated with the design of antennas for body-worn gateway devices.

A body-worn antenna needs to be flexible, lightweight, small in size, and made of materials allowing direct placement on the human or animal skin. Moreover, to ensure an adequate power transfer between devices for in-/on- or off-body communications, the antenna needs to maintain stable on-body performance and a bandwidth that covers the frequency bands of interest with an omnidirectional radiation pattern [10].

To assess the safety of body-worn antennas for health telemetry applications, the peak 1 g and 10 g specific absorption rates (SARs) and the SAR distribution on a human body phantom need to be evaluated. Obtained SAR values must be compared with limits reported in international guidelines and standards [11,12].

Several types of flexible textile-based antennas for body-worn devices, such as monopole [13,14], monopole integrated with a reflector [2], squire slot [15], loop [16], patch [17,18], logo [19], and MIMO [20] antennas, have been presented. Furthermore, antennas with polymer-based substrates such as dipoles [21], dipoles integrated with a reflector [5,10,22], monopoles combined with a metasurface [23], and monopoles and CPW-PIFA fabricated on Rogers flexible substrates [15,24,25] have been proposed for body-worn applications.

Most flexible wearable antennas reported in the literature have a high degree of isolation between the antenna and human body tissue [2,5,8,10,15,16,19,23,25] and directional radiation patterns, which make these designs unsuitable for communications between an antenna placed on the body and another one inside the body. Only a few previous studies [6,20,21,22,24,26] presented the design of body-worn antennas for in-/on- and off-body communications. In these studies, the antennas still suffered from a relatively large footprint. Another drawback is that no studies have been conducted to determine the radiation efficiency when the antennas are on a human body phantom.

Several techniques to miniaturize wearable antennas have been proposed. These techniques can be divided into two categories: (1) techniques that include a change in the geometrical shape of the antenna [27] or that use a meander [28] or a fractal shape [29]; (2) material loading techniques, using a substrate with a high dielectric constant [30] and low loss tangent or using a substrate from magneto-dielectric materials that have a relative permittivity and permeability greater than unity [31]. The most extensively used technique in antenna miniaturization is shaping the antenna geometry using fractal shapes [32]. The Koch curve, Hilbert curve, Peano curve, and Sierpiński triangle are some of the most used geometries in antenna design [33].

Several wireless technologies have been identified for wireless communication be-tween wearable devices used in healthcare for monitoring and diagnostic purposes. The most used wireless technologies include Bluetooth, Wi-Fi, and Zigbee [34]. Generally, the range of frequencies associated with wearable devices used in healthcare covers the band between 2.4 GHz and 2.48 GHz (i.e., industrial, scientific, and medical (ISM 2.4 GHz) band). Moreover, a new medical BAN (MBAN) band, which operates from 2.36 to 2.4 GHz, has been introduced for such applications [35]. Therefore, there is an increasing demand for a flexible and compact antenna with a bandwidth from 2.36 to 2.48 GHz and an omnidirectional radiation pattern for wearable devices.

In this paper, a new highly miniaturized wearable antenna is proposed for in-/on- and off-body wireless communications. To significantly reduce the size of the antenna, the quadratic Koch curve, also known as the eight-segment Koch curve or Minkowski curve, is used. The novelty in our design also lies in the fact that the proposed antenna is developed on a new polymer substrate to achieve comfortable mounting on the human body. Compared with other reported flexible antennas, the proposed antenna placed on a human body model demonstrates smaller dimensions, better radiation efficiency, and maximum gain. The antenna design steps, performance (for two scenarios: in- to on-body and on- to off-body communications), and SAR distributions on a human body phantom are also presented.

## 2. Methods, Models, and Materials

### 2.1. Methods

During the antenna’s design, analysis, and optimization process, the finite-difference time-domain (FDTD) simulation software xFDTD (xFDTD, Remcom Inc., State College, PA, USA) was used. The FDTD method was chosen because it enables detailed modeling of both antennas and human models [36]. Simulation parameters in all FDTD calculations were as follows: a nonuniform mesh having a fine-cell size of 0.5 mm and coarse-cell size of 3 mm; absorbing boundaries—perfectly matched layer; sources—a Gaussian (pulse width of 32 timesteps) for the broadband and a sinusoidal for the single-frequency simulation. The number of timesteps used for an accurate assessment of the antenna performance on human body models was 25,000 (on Phantom 1) and 50,000 (on Phantom 2). The 12-field-component approach was selected to calculate the SAR.

### 2.2. Numerical Models

Two numerical homogenous human body models (called Phantom 1 and Phantom 2) that represent the human body in all FDTD simulations were developed. Phantom 1 (dimensions 61.5 mm (x), 10 mm (y), and 46 mm (z)) was used during the antenna design and optimization process to reduce the simulation time and computational memory. The antenna performance and SAR distributions were investigated with the help of Phantom 2 (dimensions 180 mm (x), 150 mm (y), and 120 mm (z)) described in [16]. The electromagnetic properties of the numerical models were the same as those of the experimental phantom from Table 1.

Moreover, to investigate in- and off-body communication channels, two numerical models of dipole antennas (Dipole 1 and Dipole 2) were developed. The first one (Dipole 1: length of 39.5 mm and a radius of 2 mm) was designed for in-body wireless communications, and was placed inside Phantom 2. Dipole 2 (length of 54.5 mm and a radius of 2 mm) was designed for off-body wireless communications and was placed in free space. The dipoles were modeled as perfect electric conductors (PECs).

Lastly, we developed several numerical models of the body-worn antenna during the design and optimization process described in the next section.

### 2.3. Experimental Models

A flat semisolid 2/3 muscle-equivalent phantom (dimensions 180 mm × 150 mm × 120 mm) was prepared adapting the recipe from [37]. After 24 h solidification, the electromagnetic properties (EM) of the phantom were measured using the resonant perturbation method at 2.565 GHz at room temperature. Table 1 presents the real part (ε_r_’) of the complex relative permittivity, electrical conductivity (σ), and density (ρ) of the solid mixture.

Furthermore, two dipole antennas (Experimental Dipole 1 and Experimental Dipole 2) were fabricated, to experimentally investigate the in- and off-body communication channels. The dimensions of the experimental dipoles were the same as those of the numerical models described in the previous subsection.

### 2.4. Materials

For the antenna substrate, a composite, composed of a mixture of natural rubber and TiO_2_ as a filler (NR-TiO_2_), was fabricated according to the procedure outlined in [10,38] and then was vulcanized to form a plate with dimensions 150 mm × 150 mm × 2.5 mm.

After the vulcanization, the EM properties of the composite were measured using the resonant perturbation method at 2.565 GHz. The results are summarized in Table 1. The measured values of ε_r_’, σ, and ρ were implemented in the numerical models of the antenna (to the substrate) and used during the design and optimization process described in the next section.

Brass foil (thickness 0.05 mm) was employed to realize the conductive parts of the proposed antenna.

## 3. Antenna Design and Numerical Studies of the Antenna Performances

### 3.1. Antenna Design Concept

The design goal is to create a flexible body-worn antenna with minimal volume that maintains adequate radiation and SAR lower than the limits reported in [11,12] suitable for in-/on- and off-body wireless communications in two frequency ranges of 2.36–2.4 GHz MBAN) and 2.4–2.4835 GHz (ISM 2.4 GHz).

The design and optimization were carried out on a human body phantom (Phantom 1) since the antenna is intended to operate on a human or animal body. This phantom was used because it has a small size; thus, simulations run quickly.

**Figure 1:**
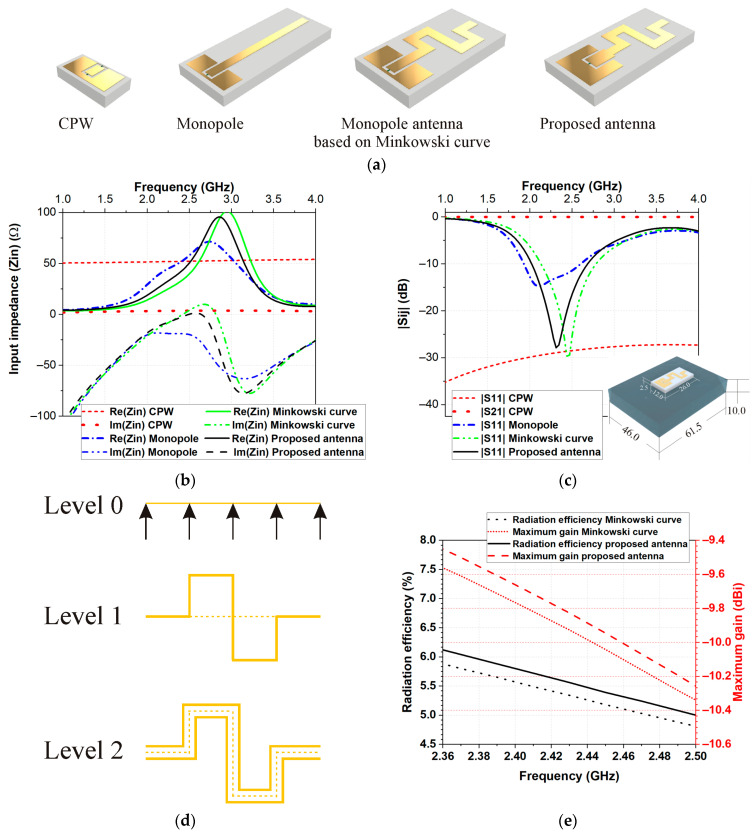
Design process: (**a**) numerical models of CPW and different configurations of the antenna during the design process, (**b**) input impedance, (**c**) magnitudes of the reflection |S_11_| and transmission |S_21_| coefficient, (**d**) generation of the antenna using the quadratic Koch curve, also known as the eight-segment Koch curve or Minkowski curve, and (**e**) comparison of the radiation efficiency and maximum gain of the proposed antenna and monopole antenna based on Minkovski curve when the antennas were placed on Phantom 1.

First, a coplanar waveguide (CPW) transmission line on a flexible polymer-based NR-TiO_2_ substrate was designed (Figure 1a). The CPW was optimized to provide the best combination of small dimensions and characteristic impedance (approaching the 50 Ω value (±10%)) in the frequency bands of interest. Figure 1b,c show the frequency dependence of the impedance, as well as magnitudes of the reflection |S_11_| and transmission |S_21_| coefficient.

In the second step, a rectangular monopole (length 29.5 mm) was designed as a radiator, considering its advantages of simple structure and wide bandwidth. The reflection coefficient magnitudes and input impedance at different stages of antenna design are presented in Figure 1. As can be seen in Figure 1b, the monopole antenna exhibited resonance around 2.1 GHz.

**Figure 2:**
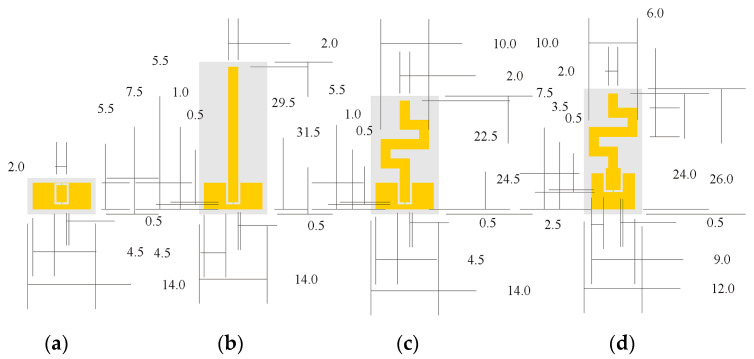
A top view of configurations of the CPW and antennas during the design process: (**a**) coplanar waveguide; (**b**) monopole antenna; (**c**) monopole antenna based on the Minkowski curve; (**d**) proposed antenna. All dimensions are in mm.

In the next step, to reduce the overall height of the antenna, we used the quadratic Koch curve, also known as the eight-segment Koch curve or Minkowski curve. First, we divided the straight line (representing the monopole length—Level 0) into four equal parts. After that, we replaced the two middle segments of the line (Level 1) with a simple figure also called a generator of the curve, as shown in Figure 1d. Lastly, the resulting curve was used as a base to create a monopole based on the Minkowski curve (Level 2), as shown in Figure 1d. The overall height of the monopole antenna based on the Minkowski curve was reduced to 17 mm (Figure 2). Comparing the reflection coefficient magnitude and input impedance of the monopole antenna based on the Minkowski curve with those of the monopole, we can see that the real part increases, while the imaginary part of the input impedance tends toward values close to zero in the frequency range of 2.2 GHz to 2.8 GHz.

Lastly, to achieve the impedance matching in the desired frequency bands with maximum possible radiation efficiency, we tuned the geometrical dimensions of CPW and monopole antenna based on the Minkowski curve, as shown in Figure 1a. The proposed new flexible miniature body-worn antenna has better radiation efficiency than the monopole antenna based on the Minkowski curve, as shown in Figure 1e.

Figure 2 shows the geometrical dimensions of the coplanar waveguide and antenna during the design and optimization process.

### 3.2. Numerical Studies of the Antenna Performances

#### 3.2.1. Reflection Coefficient

**Figure 3:**
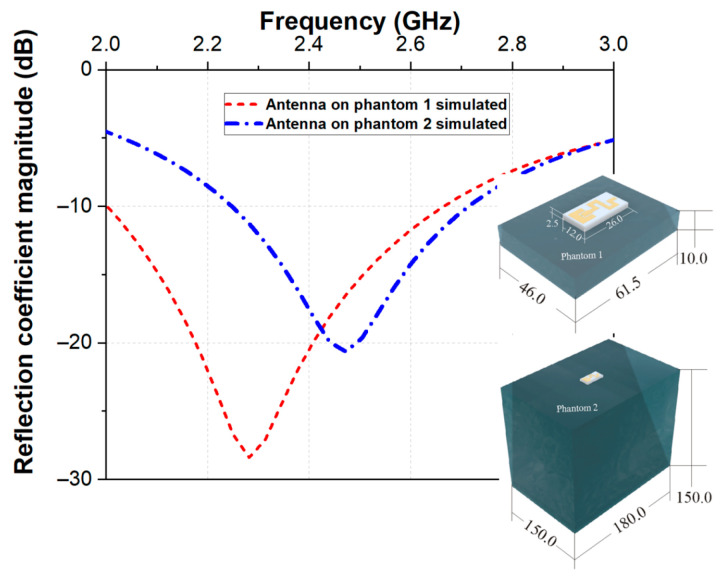
Simulated reflection coefficient magnitude versus frequency when the proposed antenna was placed on the Phantom 1 and Phantom 2.

Figure 3 displays the simulated reflection coefficients when the proposed antenna was placed on Phantom 1 and Phantom 2. Looking at the reflection coefficients, it is clear that the antenna exhibited bandwidth from 2.0 to 2.65 GHz (on Phantom 1) and from 2.25 GHz to 2.70 GHz (on Phantom 2) at |S_11_| < −10 dB covering MBAN and ISM. A shift of the resonance frequency from 2.28 GHz (on Phantom 1) to 2.47 GHz (on Phantom 2) was also observed. Observed differences are due to the difference in dimensions between Phantom 1 and Phantom 2.

#### 3.2.2. Radiation Characteristics

**Figure 4:**
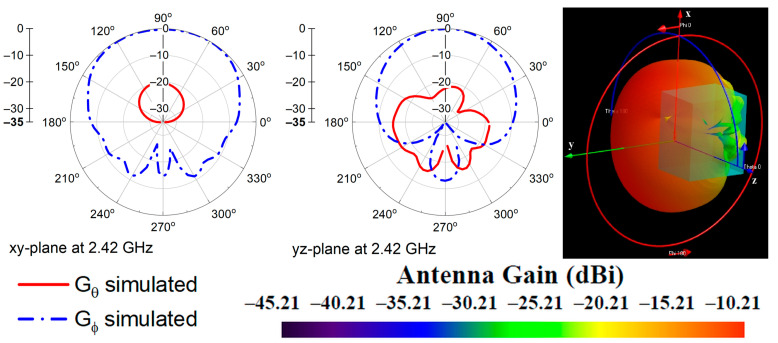
Simulated 2D normalized radiation patterns in *xy*- and *yz*-plane, and 3D radiation pattern of the proposed antenna on Phantom 2.

The radiation characteristics of the antenna were investigated across the bandwidth of 2.36–2.5 GHz when the antenna was placed on Phantom 2. Figure 4 shows the simulated radiation patterns at 2.42 GHz, in the *xy*- and *yz*-plane. The radiation patterns at frequencies from 2.36 GHz to 2.5 GHz showed similar behavior.

#### 3.2.3. Maximum Gain and Radiation Efficiency

**Figure 5:**
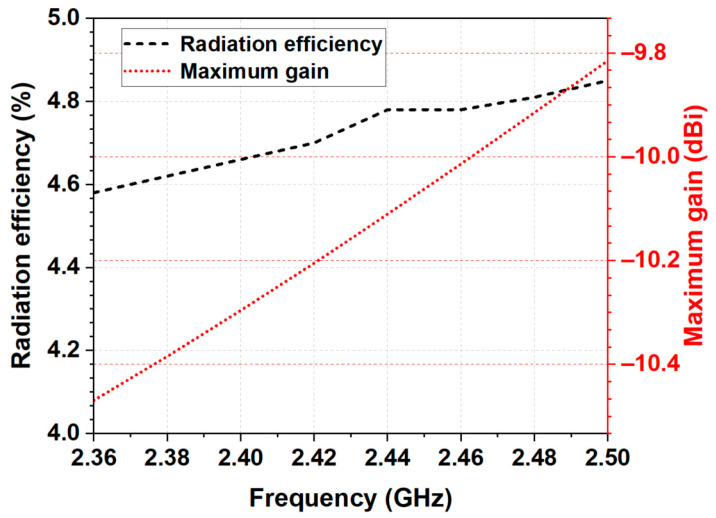
Radiation efficiency and maximum gain of the proposed antenna on Phantom 2 (shown in Figure 3).

The simulated maximum gain is given in Figure 5. As seen, the maximum realized gain varied between −10.5 dBi and −9.8 dBi and increased with frequency. The negative antenna gain is due to the power absorbed in the homogeneous phantom. Moreover, the antenna showed stable radiation efficiency (between 4.6% and 4.83%) in the frequency range of 2.36 to 2.5 GHz, which is appropriate for health telemetry applications.

#### 3.2.4. Transmission Performances

**Figure 6:**
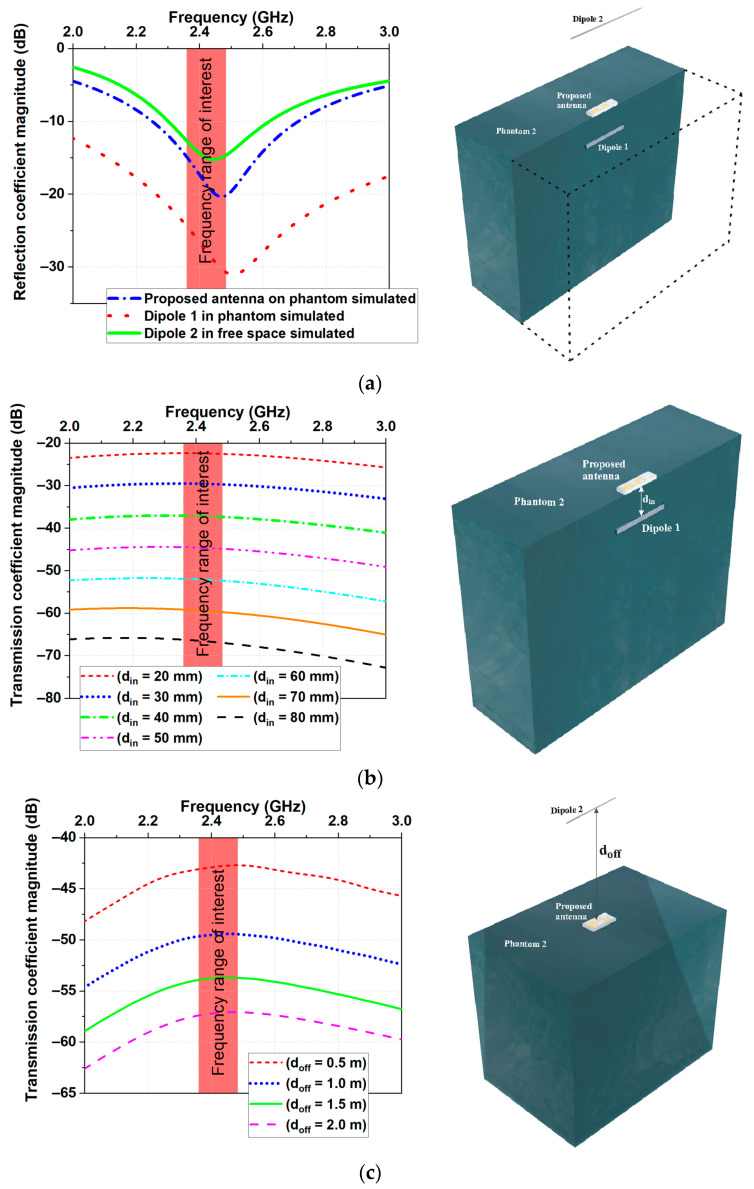
Simulated (**a**) reflection coefficients, (**b**) transmission coefficient for in- to the on-body scenario, and (**c**) transmission coefficient for on- to the off-body scenario. Illustrations of the setup for S-parameter simulations and measurements.

The capability of the proposed antenna for in- to on-body (scenario 1) and on- to off-body (scenario 2) communications was also studied. Figure 6a presents the setup for numerical studies of the transmission performances of the proposed miniaturized antenna. As seen, the setups consisted of two dipoles (Dipole 1 and Dipole 2) and a homogeneous phantom (Phantom 2). Dipole 1 was designed to resonate at 2.508 GHz when incorporated into Phantom 2, while Dipole 2 was designed to resonate at 2.444 GHz in free space. The simulated reflection coefficient magnitudes versus frequency are presented in Figure 6a for the two dipoles.

For the first scenario (in- to on-body communications), the miniaturized antenna acted as a receiving antenna, whereas Dipole 1 acted as a transmitting one. For this case, the miniaturized antenna was fixed on the surface of Phantom 2, whereas Dipole 1 was moved inside the phantom from starting distance d_in_ = 20 mm up to d_in_ = 80 mm in steps of 10 mm (see Figure 6b). The simulated transmission coefficient magnitudes are presented in Figure 6b for various locations of the in-body antenna. Results show that the transmission coefficient magnitudes were higher than −66 dB in the frequency range of interest even at a distance of 80 mm, which is an acceptable value for a typical receiver.

For the second scenario (on- to off-body communications), the miniaturized antenna acted as a transmitting antenna, whereas Dipole 2 acted as a receiving one. The simulation setup for this scenario is similar to the in- to on-body communication scenario. In this case, Dipole 2 was moved in the free space away from the phantom. The distance from the phantom surface to Dipole 2 was denoted as d_off_ (see Figure 6c). The investigations were carried out at four fixed values for d_off_ (0.5 m, 1.0 m, 1.5 m, and 2 m). The simulated transmission coefficient magnitudes are presented in Figure 6c for various locations of the off-body antenna. As expected in this scenario, the transmission coefficient magnitudes were higher than −60 dB even at a distance of 2.0 m, which satisfies the requirement of the sensitivity of standard receivers at the ISM 2.4 GHz band [23].

From the results, we can conclude that the maximum telemetry range that can be achieved from the antenna is more than 80 mm for in-body communications and more than 2 m for off-body communications.

## 4. Fabrication of the Antenna Prototype

**Figure 7:**
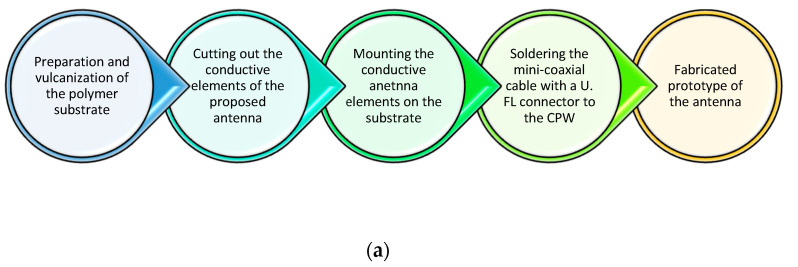

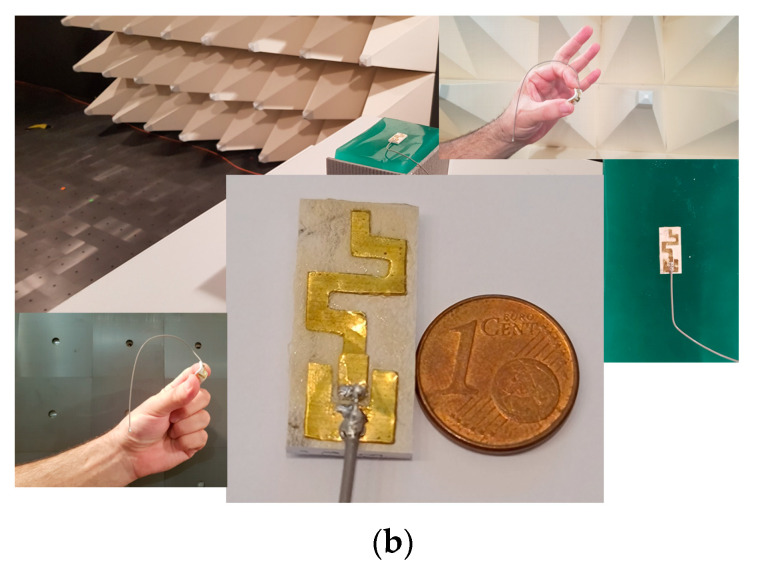
Fabrication process: (**a**) block diagram and (**b**) photographs of the fabricated antenna and experimental flat semisolid 2/3 muscle phantom.

A schematic presentation of the antenna prototype is depicted in Figure 7a. In the first step of the fabrication process, the antenna substrate was prepared by mixing natural rubber, vulcanization accelerators, compatibilizers, and filler (TiO_2_) in an open laboratory two-roll mill. The mixture was vulcanized to a plate with dimensions 150 mm × 150 mm × 2.5 mm. In the second step, the conductive elements of the proposed antenna were fabricated from brass foil using a cutting plotter. Next, the conductive elements were mounted on the substrate with liquid rubber glue. Lastly, a mini coaxial cable assembled with a U.FL connector was soldered to the CPW for measurement purposes. Photographs of the fabricated antenna and experimental flat semisolid 2/3 muscle phantom are presented in Figure 7b.

## 5. Experimental Results

**Figure 8:**
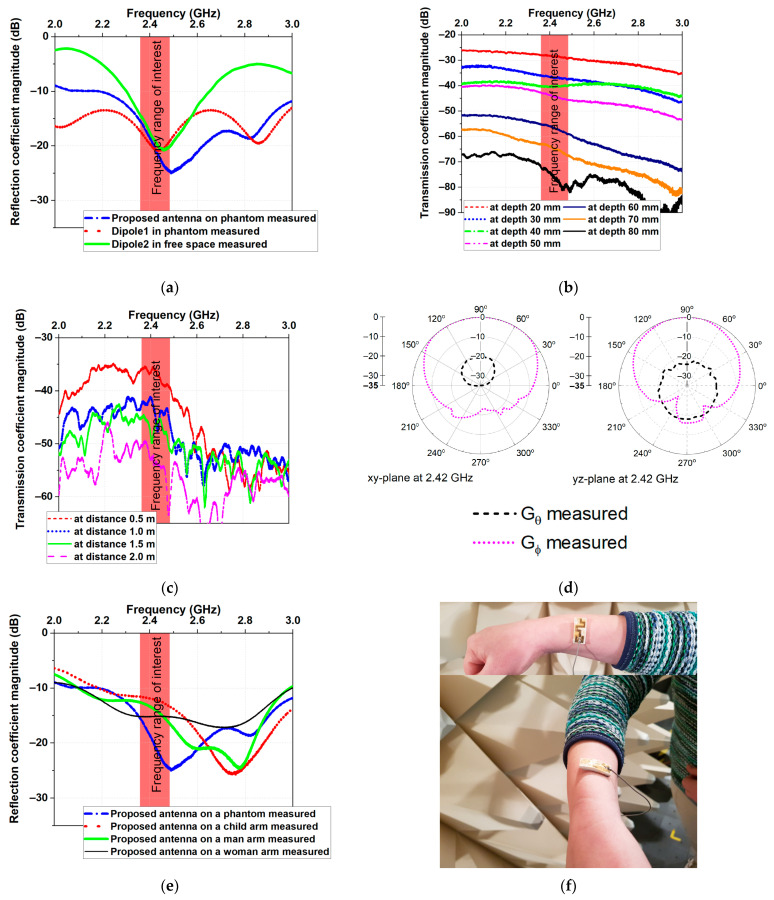
Measured (**a**) reflection coefficient magnitudes, (**b**) transmission coefficient magnitudes for scenario 1 (in- to on-body communications), (**c**) transmission coefficient magnitudes for scenario 2 (on- to off-body communications), (**d**) 2D normalized radiation patterns in *xy*- and *yz*-plane, and (**e**) reflection coefficient magnitudes antenna on human arms. (**f**) Photographs of device.

The proposed antenna was tested on the fabricated experimental flat semisolid 2/3 muscle phantom to validate the antenna design and performance. The reflection and transmission coefficient magnitudes were measured using a Tektronix TTR500 series TTR503A vector network analyzer (100 kHz–3 GHz frequency range). Measurements were performed in the frequency range of 2.0 GHz to 3.0 GHz with 20,001 resolution points. Figure 8a depicts the measured reflection and transmission coefficients of Dipole 1, Dipole 2, and the proposed antenna according to the test setup presented in Figure 6a. Good agreement was obtained between simulations (Figure 6a) and measurements (Figure 8a).

Figure 8b,c display the measured transmission coefficients for scenario 1 (in- to on-body communications) and scenario 2 (on- to off-body communications). Comparing the transmission coefficients presented in Figure 6 and Figure 8, we can see that the measured results agreed well with the simulations in the frequency range of interest.

The proposed antenna’s radiation patterns were measured in a semi-anechoic chamber, and the results are presented in Figure 8d. The measured radiation patterns of the proposed antenna agreed well with the simulated results in both planes.

The effects of bending on the antenna performance were also investigated. The |S11| and bandwidth were evaluated when the prototype was bent over the arms of a child, man, and woman. As shown in Figure 8e, the antenna bandwidth did not show any detectable change after bending. A shift in the resonance frequency was observed. Hence, we can conclude that bending had a minor effect on the antenna impedance matching.

Moreover, the elongation of the polymer composite evaluated according to ISO 37:2018 [39] was 1.7 MPa.

## 6. Specific Absorption Rate

Lastly, the impact of the wearable antenna on the human body was investigated by calculating the specific absorption rate. In all simulations, the flexible body-worn miniature antenna was on the surface of Phantom 2.

**Figure 9:**
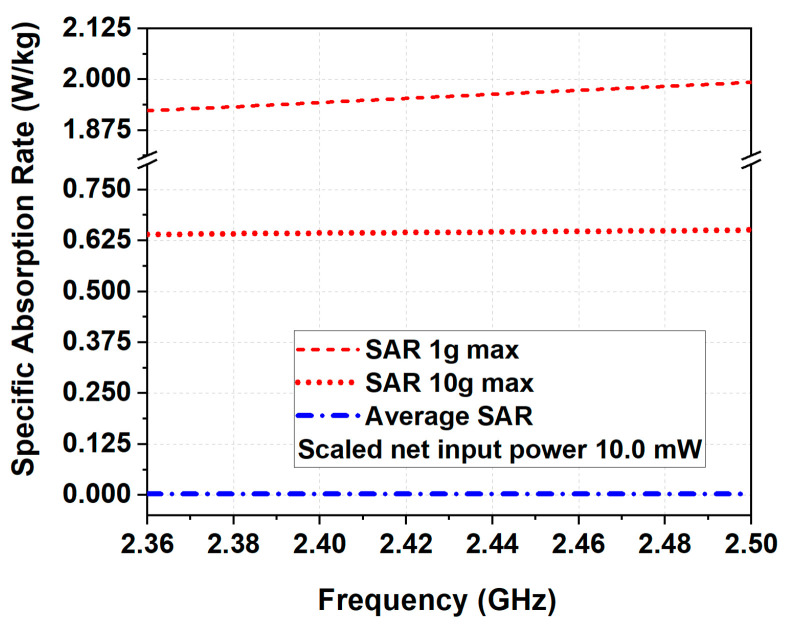
Simulated 1 g, 10 g, and averaged SAR for the proposed antenna mounted on the surface of Phantom 2.

Results show that the highest 1 g (1.98 W/kg per 10 mW of antenna delivered power) and 10 g (0.62 W/kg per 10 mW of antenna delivered power) SAR values occurred at 2.48 GHz (see Figure 9). According to the specification provided by the ICNIRP [11], SAR values must be no greater than 2 W/kg averaged over 10 g of tissue. Consequently, the 10 g SAR was below the European threshold of 2 W/kg and the limit proposed in [11].

**Figure 10:**
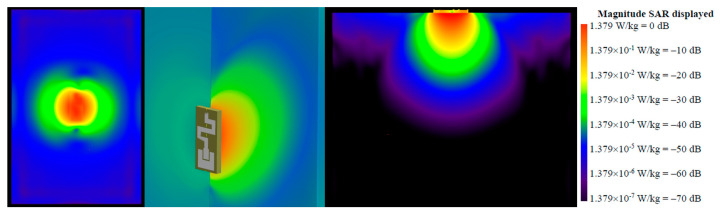
SAR distribution at 2.48 GHz.

Figure 10 displays the SAR distribution at 2.48 GHz. It can be observed that the maximum SAR occurred at the surface underneath the antenna.

## 7. Comparison

Lastly, to demonstrate the advantages of the proposed flexible body-worn miniature antenna for remote monitoring of human health, its performance metrics are compared to metrics of the other previously reported antennas for in- to on-body and on- to off-body communications. The results are presented in Table 2. It can be seen that the proposed antenna had smaller dimensions than described in [6,21,22,24,26,40,41], better radiation efficiency than described in [26,40], and higher maximum gain than described in [40]. In addition, the proposed antenna had the same maximum 1 g SAR compared to the antenna in [22]. However, the proposed antenna has outstanding features of a simple fabrication process and a low-cost natural rubber substrate.

## 8. Conclusions

A miniature flexible body-worn antenna was proposed and analyzed for remote monitoring of human health applications. The antenna achieved more than 4.5% on-body radiation efficiency over the targeted frequency bands. Moreover, the proposed antenna realized a maximum telemetry range of more than 80 mm for in-body communications and more than 2 m for off-body communications. These results, in addition to the antenna’s compact dimensions (12 mm × 26 mm × 2.5 mm) and the low manufacturing price, make the proposed antenna an ideal candidate for health telemetry applications.

## Figures and Tables

**Table 1:** Material properties of the antenna substrate (NR-TiO_2_) and 2/3 muscle-equivalent phantom measured at 2.565 GHz.

	NR-TiO_2_	2/3 Muscle-Equivalent Semisolid Phantom
ε_r_’	3.1644	43.0
σ (S/m)	0.000816	2.2
ρ (kg/m^3^)	940	1166

**Table 2:** Comparison of the metrics of the different body-worn antennas with the proposed antenna.

References	Rad. Eff. * (%)	Max. Gain ** (dBi)	Antenna Dimensions (λ ***)	Frequency Range, GHz Simulated	Max. 1 g SAR, W/kg ****
[6]	NA	NA	0.286λ × 0.006λ	2.4–2.48; 4–10.6	NA
[21]	NA	NA	0.294λ × 0.620λ	1–4; 3–5	NA
[22]	NA	−2.10	0.473λ × 0.327λ × 0.0001λ	1.4–2.6; 3–5.9	1.96
[24]	NA	NA	0.327λ × 0.327λ	2.4–3.5; 4.5–6	NA
[26]	3	NA	0.727λ × 0.490λ × 0.171λ	3.75–4.25	NA
[40]	0.5	−18	0.218λ × 0.229λ × 0.005λ	0.401–0.406; 2.4–2.48	NA
[41]	NA	NA	0.245λ × 0.286λ × 0.001λ	1.9–2.2	NA
Proposed antenna	4.76	−9	0.098λ × 0.212λ × 0.020λ	1.98–2.8	1.96

* Rad. Eff—radiation efficiency when the antenna is placed on a human body model. ** Max. Gain—maximum gain when the antenna is placed on a human body model. *** λ—wavelength in free space at 2.45 GHz. **** Max. 1 g SAR—maximum 1 g SAR at 2.45 GHz when the antenna is on a human body model. All results were normalized to net input power of 10 mW. NA—not available.

## Data Availability

The data presented in this study are available on request from the corresponding author.
